# Manufacture of Radio Frequency Micromachined Switches with Annealing

**DOI:** 10.3390/s140101680

**Published:** 2014-01-17

**Authors:** Cheng-Yang Lin, Ching-Liang Dai

**Affiliations:** Department of Mechanical Engineering, National Chung Hsing University, Taichung 402, Taiwan; E-Mail: g099061077@mail.nchu.edu.tw

**Keywords:** micromachined switches, annealing, post-process

## Abstract

The fabrication and characterization of a radio frequency (RF) micromachined switch with annealing were presented. The structure of the RF switch consists of a membrane, coplanar waveguide (CPW) lines, and eight springs. The RF switch is manufactured using the complementary metal oxide semiconductor (CMOS) process. The switch requires a post-process to release the membrane and springs. The post-process uses a wet etching to remove the sacrificial silicon dioxide layer, and to obtain the suspended structures of the switch. In order to improve the residual stress of the switch, an annealing process is applied to the switch, and the membrane obtains an excellent flatness. The finite element method (FEM) software CoventorWare is utilized to simulate the stress and displacement of the RF switch. Experimental results show that the RF switch has an insertion loss of 0.9 dB at 35 GHz and an isolation of 21 dB at 39 GHz. The actuation voltage of the switch is 14 V.

## 1. Introduction

Radio frequency switches are applied in wireless communication systems [1]. Compared with solid-state RF switches, the benefits of micromachined RF switches are low insertion loss, excellent isolation, and high linearity at microwave frequencies [2,3]. Recently, several micromachined switches have been fabricated using microelectromechanical system (MEMS) technology. For instance, Czaplewski *et al.* [4] presented a RF MEMS switch design that improved lifetimes in cycled switches. The implementation of RuO_2_-Au contact metallurgy into the switch improved the lifetime of the switch, which was cycled to 10 billion cycles with a resistance of less than 4 Ω. The insertion loss and isolation of the RF switch were 0.4 dB and 28.0 dB at 10 GHz, respectively. Zhu *et al.* [5] employed the MetalMUMPs process to fabricate a lateral dc-contact RF MEMS switch for ultrabroadband applications. A bidirectional cascaded electrothermal actuator was designed to drive the switch. The RF switch had an insertion loss of 0.5 dB at 40 GHz and an isolation of 22.5 dB at 40 GHz. Park *et al.* [6] used the sacrificial bulk micromachining process to manufacture a RF MEMS switch for 24 GHz radar applications. The switch was actuated by comb-drive actuators, and it was a capacitive shunt type. The actuation voltage of the RF switch was 25 V. The RF switch had an insertion loss of 0.29 dB at 24 GHz and an isolation of 30.1 dB at 24 GHz. Kügeler *et al.* [7] proposed a silicon based micromachined switch with piezo-electrically actuated elements. The switch consisted of two piezoelectric activated beams with a coplanar waveguide (CPW). The clamped-clamped beams were established by a thin PZT film between thin Pt electrodes on top of a SiO_2_ layer, and the CPW was made by the electroplated copper. The switch had an actuation voltage of 10 V and an isolation of 20 dB at 15 GHz. Chang *et al.* [8] utilized surface micromachining process to fabricate a micromachined microwave switch on a GaAs substrate. The process used the lift-off technique to pattern the CPW lines of Cr/Au, followed by defining the actuator structure layers of Al/Cr deposited by electron beam evaporation. The insertion loss of the switch was 0.2 dB at 10 GHz, and its isolation was 17 dB at 10 GHz. The actuation voltage of the switch was 26 V. Zheng *et al.* [9] developed an RF MEMS membrane switch on a GaAs substrate using surface micromachining process. The RF switch contained CPW lines of AuGeNi/Au, a dielectric layer of SiN and a membrane of Au. The switch had an isolation of 42 dB at 24.5 GHz and an insertion loss of 0.25 dB at 25.6 GHz. The actuation voltage of the switch was 17 V. In this work, we uses the commercial CMOS process to manufacture a micromechanical RF switch, and its fabrication is easier than Czaplewski *et al.* [5], Park *et al.* [6], Kügeler *et al.* [7], Chang *et al.* [8], and Zheng *et al.* [9].

The commercial CMOS process has been employed to manufacture various microdevices [10–14]. Microdevices fabricated by this process usually need a post-process step to release suspended structures [15–17] and to add functional materials [18,19]. The CMOS microdevices have a potential for integration with circuitry on-a-chip [20]. In this work, we develop a micromechanical RF switch using the commercial CMOS process. The structure of the RF switch contains a membrane, eight springs, and CPW lines. The stress and displacement of the RF switch are simulated using the FEM software CoventorWare. The electrical properties of the switch are simulated using the Agilent Advanced Design System (ADS). A wet etching post-process step is used to etch the sacrificial silicon dioxide layer, and to release the suspended structures of the switch. The membrane of the switch occur a deformation due to the residual stress. To improve the residual stress of the switch, an annealing process is applied to the switch. The switch is a capacitive shunt type actuated by the electrostatic force.

## 2. Structure of the RF Switch

**Figure 1.**
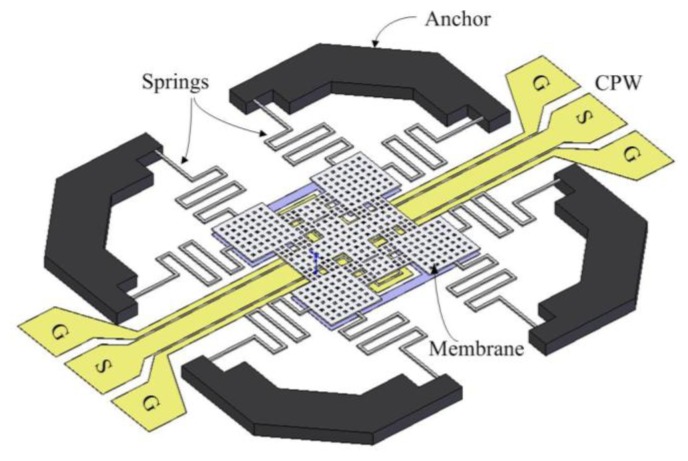
Structure of RF micromachined switch.

**Figure 2.**
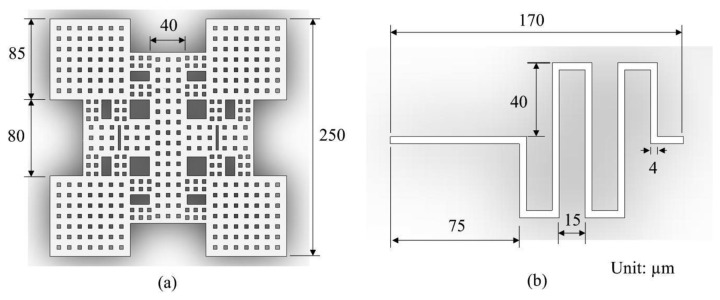
Dimensions of (**a**) membrane; and (**b**) springs.

Figure 1 shows the schematic structure of the micromachined RF switch. The RF switch is composed of a membrane, eight springs, CPW transmission lines, and anchors. The membrane is supported by eight springs. The dimensions of the membrane are shown in Figure 2a. Figure 2b shows the dimensions of a spring, and all springs have the same dimensions. The thickness of springs and membrane is 1 μm. The CPW transmission lines contained ground (G), signal (S) and ground (G) lines locate under the membrane. The width and thickness of the ground lines are 110 μm and 0.53 μm, respectively. The width and thickness of the signal line are 35 μm and 0.53 μm, respectively. The space between the signal and ground lines is 3.1 μm. The RF switch is a capacitive shunt type actuated by electrostatic force. When there is no applied voltage, the membrane stays in the up position. The switch is at the “on” state, and the RF signal propagates in the signal line of CPW. When applying an actuation voltage to the switch, the membrane actuated by the electrostatic force stays in the down position. The switch is at the “off” state, and the RF signal propagated in the signal line is coupled to the ground lines.

The insertion loss and isolation of the RF switch depend on the flatness of membrane. To obtain excellent membrane flatness, this study adopts an annealing process to improve the residual stress of the membrane. The insertion loss and isolation of this work exceed that of the previous works [10,21]. In addition, the membrane has many etching holes as shown in Figure 2a, in order to reduce the etching time during the post-process. The actuation voltage of the RF switch depends on the stiffness of springs. To reduce the stiffness of springs and obtain a lower actuation voltage, the springs are designed as S-shape.

**Figure 3.**
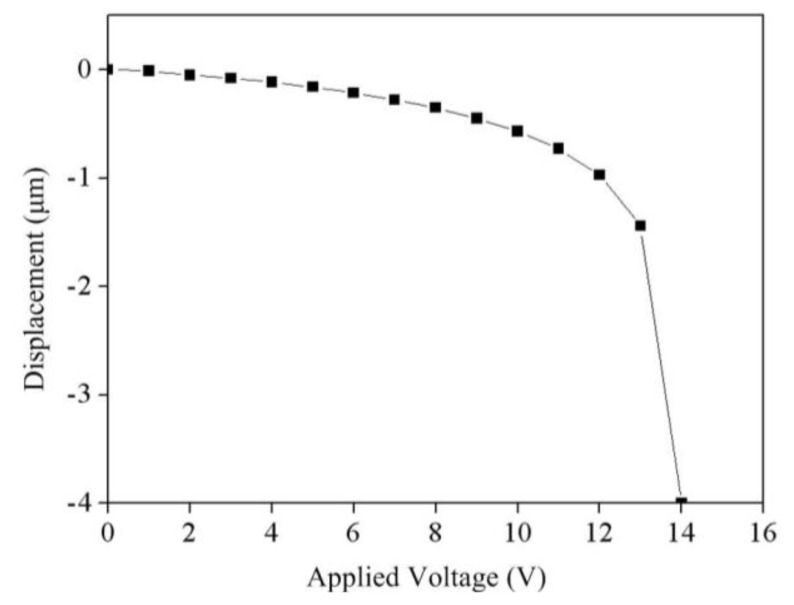
Relation between actuation voltage and membrane displacement.

**Figure 4.**
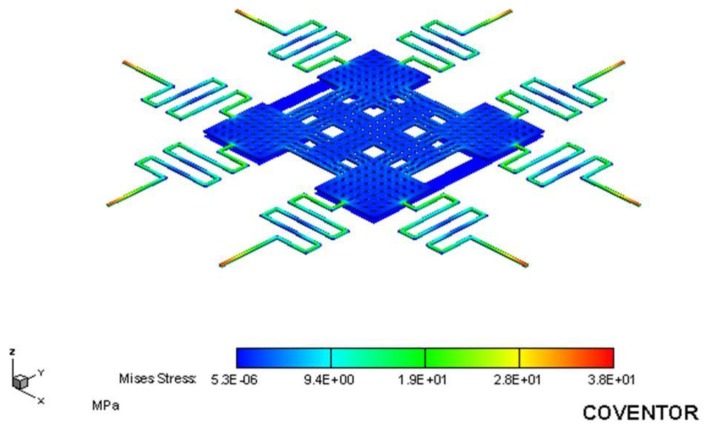
Stress distribution of the RF switch.

The FEM software CoventorWare was used to simulate the stress and displacement of the RF switch. According to the structure as shown in Figure 1 and the dimensions as shown in Figure 2, the model of the RF switch is established. The triangular element is adopted to mesh the model of the switch. The material of the switch is aluminum. The material properties of aluminum are mass density, 2,679 kg/m^3^; Young's modulus, 70 GPa; Poisson's ratio, 0.3 [22]. Figure 3 shows the relation between the membrane displacement and actuation voltage for the RF switch. The space between the membrane and the CPW lines is 4 μm. In this simulation, the actuation voltage changes from 0 to 14 V. The membrane displacement is 1.3 μm when applying an actuation voltage of 13 V and the displacement is 4 μm when applying an actuation voltage of 14 V. Therefore, the pull-in of the RF switch is about 14 V. Figure 4 displays the stress distribution of the switch with an actuation voltage of 14 V.

The simulation results reveal that the maximum stress of the RF switch is 38 MPa that locates at the anchored end of the springs. The maximum stress of the switch is below the yield strength of aluminum (124 MPa). Thereby, the deformation of the switch operates in the elastic range.

**Figure 5.**
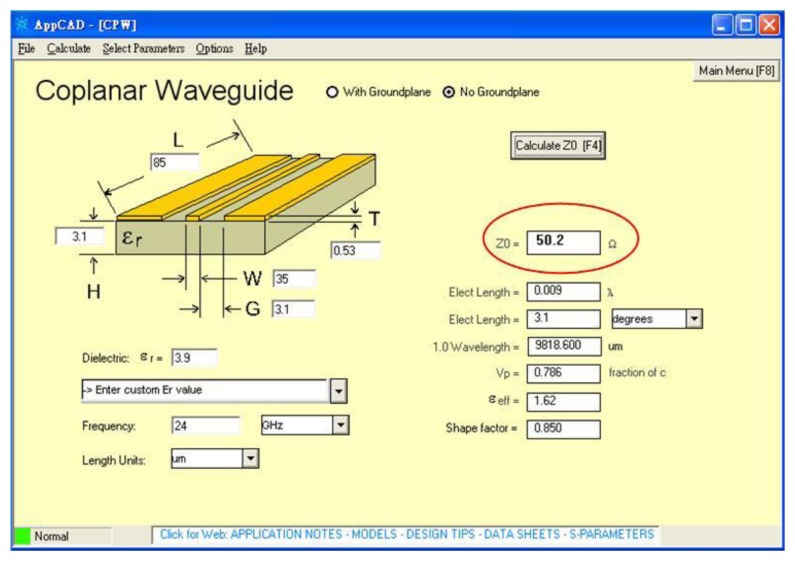
Simulation of characteristic impedance for the CPW.

The characteristic impedance of the CPW lines was evaluated using the Agilent CAD tool. Figure 5 demonstrates the evaluated results of the characteristic impedance for the CPW lines. In this evaluation, the width and thickness of the signal line are 35 μm and 0.53 μm, respectively. The space between the signal and ground lines is 3.1 μm. The results reveal that the characteristic impedance of the CPW is 50.2 Ω, and the value matches the impedance of 50 ΩΩin the network analyzer. This represents that the incident electromagnetic wave on the switch has a small return loss.

**Figure 6.**
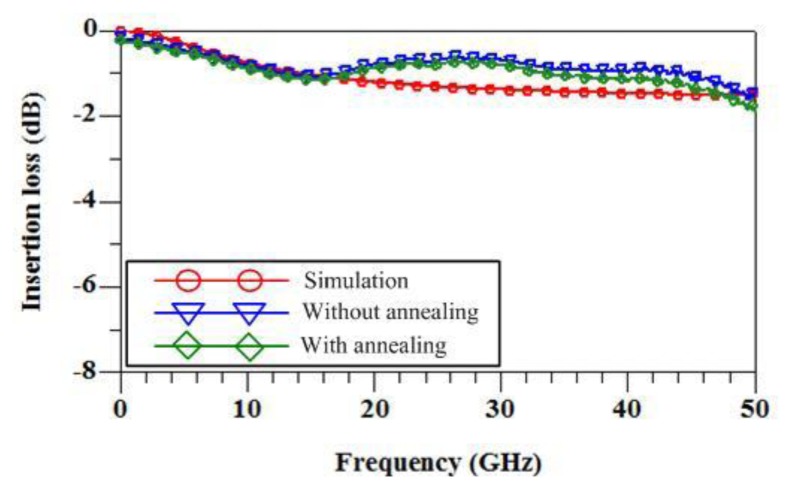
Insertion loss of the RF switch.

**Figure 7.**
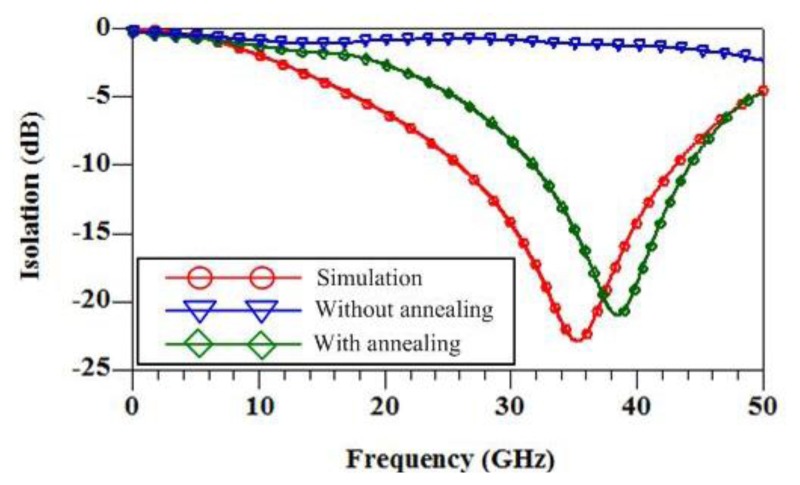
Isolation of the RF switch.

The electrical properties of the RF switch are simulated using the Ansoft Q3D extractor and the Agilent ADS. The electrical parameters of the switch in accordance with the dimensions as shown in Figure 2 are extracted using the Ansoft Q3D extractor [21]. The extracted results show that the capacitance between the membrane and the signal line is 0.04 pF; the inductance of the membrane and springs is 0.23 nH; the resistance of the membrane and springs is 1.8 Ω; the insulated capacitance under the CPW lines is 48 fP; the resistance of silicon substrate is 250 Ω; the capacitance of silicon substrate 30 fF. The electrical parameters are inputted into the Agilent ADS, and the insertion loss and the isolation of the RF switch can be obtained. Figure 6 displays the simulation results of insertion loss for the switch in the “on” state. The results reveal that the RF switch has an insertion loss of 1.1 dB at 35 GHz. Figure 7 presents the simulation results of isolation for the switch in the “off” state. The results show that the isolation of the RF switch is 23 dB at 35 GHz.

## 3. Fabrication of RF Switch

**Figure 8.**
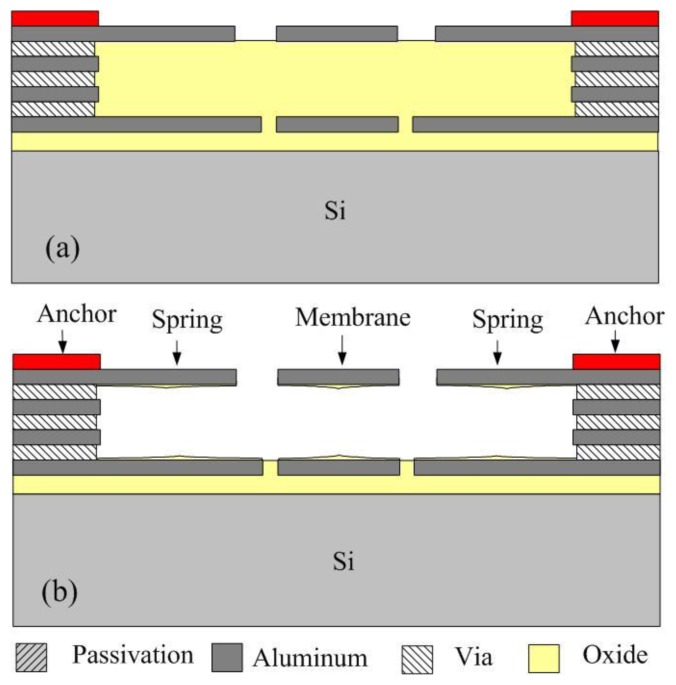
Fabrication flow of the RF switch: (**a**) after the CMOS process; and (**b**) after the post-process.

The 0.35 μm CMOS process of the Taiwan Semiconductor Manufacturing Company (TSMC, Taipei, Taiwan) was employed to fabricate the RF switch. The process flow of the RF switch was illustrated in Figure 8. Figure 8a shows the cross-sectional view of the RF switch after the CMOS process.

**Figure 9.**
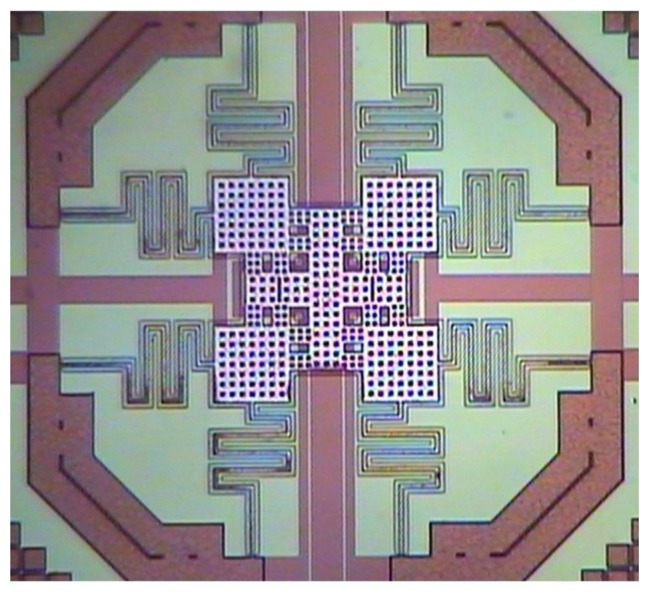
Optical image of the RF switch after the post-process.

The material of springs and membrane was aluminum. The anchors were the laminated structures of aluminum and stack-via layers. The material of via layers was tungsten. A sacrificial silicon dioxide layer was located under the membrane and springs. The switch needed a post-process to etch the sacrificial silicon dioxide layer and to release the membrane and springs [23–25]. Figure 8b shows the cross-sectional view of the RF switch after the post-process. A wet etching of BOE (buffer oxide etch) solution was used to remove the sacrificial silicon dioxide layer and to obtain the suspended membrane and springs. The etching rate is about 960 Å/min. Figure 9 demonstrates the optical image of the RF switch after the post-process.

## 4. Results

**Figure 10.**
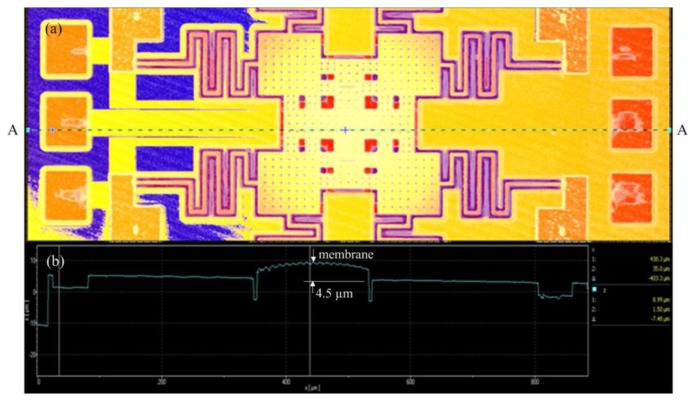
RF switch without annealing: (**a**) top view image; (**b**) profile along AA cross-section.

The membrane of the RF switch produced a deformation because of residual stress. To characterize the deformation of the membrane, a white light interferometer was used to measure the profile of the RF switch. Figure 10a displays the image of top view for the RF switch.

**Figure 11.**
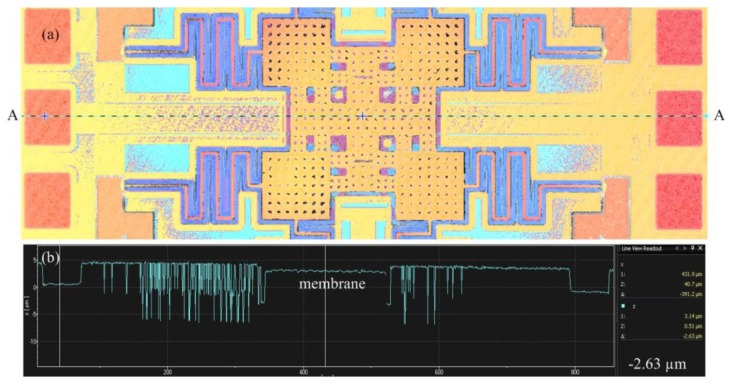
RF switch with annealing: (**a**) top view image; (**b**) profile along AA cross-section.

**Figure 12.**
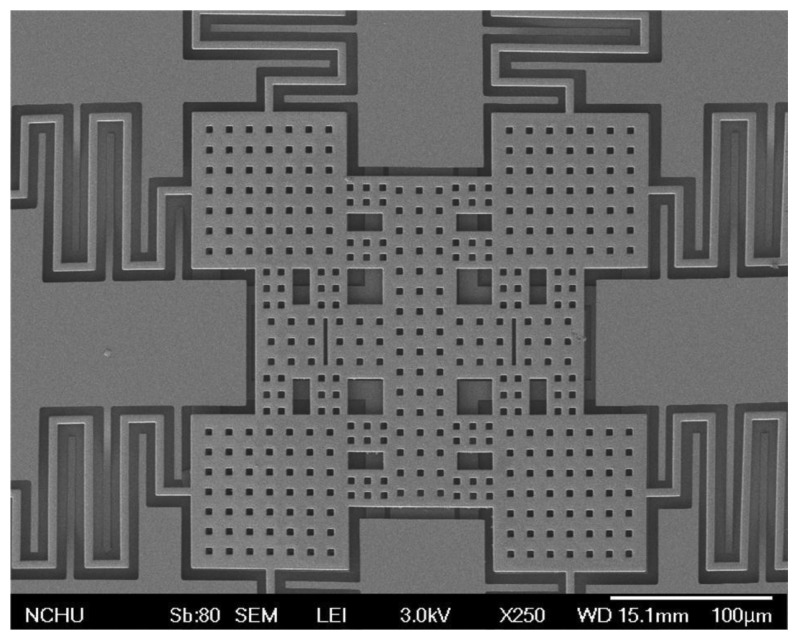
SEM image of the membrane with annealing.

Figure 10b presents the profile of the RF switch along AA cross-section (Figure 10a). The measurement results showed that the middle position of the membrane had a deformation of 4.5 μm. The residual stress influences the performance of the RF switch. To improve the residual stress, an annealing process was applied to the RF switch. The switch was set into a furnace with annealing at 300 °C for 30 min. The Figure 11a depicts the image of top view for the RF switch with the annealing. Figure 11b shows the profile of the RF switch with the annealing along AA cross-section (Figure 11a). The results showed that the membrane changed to flatness. Figure 12 depicts a SEM image of the membrane of the RF switch with the annealing, and the membrane has an excellent flatness.

The performances of the RF switch were measured using an Agilent 8510C network analyzer and a Cascade probe station. The S-parameters of the switch were obtained by a de-embedded procedure to remove the undesired pad parasitic [26]. The RF switch was in the unactuated state or “on” state when there was no applied voltage. The Agilent 8510C network analyzer measured the S-parameters of the switch in the range 0–50 GHz. Figure 6 shows the measurement results of insertion loss for the RF switch in the unactuated state. The results revealed that the switch had an insertion loss of 0.9 dB at 35 GHz. A comparison to the simulation results as shown in Figure 6, the measurement results of insertion loss are in good agreement with the simulation results of insertion loss. When applying an actuation voltage of 14 V, the RF switch was in the actuated state or “off” state. Figure 7 presents the measurement results of isolation for the RF switch in the actuated state. The results showed that the RF switch had an isolation of 21 dB at 39 GHz. As shown in Figure 7, the simulation results of isolation is 23 dB at 35 GHz. The difference between the simulation and measurement results of isolation was 2 dB at resonance frequency.

The RF switch without annealing was also tested. As shown in Figure 6, the results revealed that the insertion loss of the RF switch without annealing was 1.2 dB at 35 GHz. Figure 7 presents the isolation of the RF switch without annealing. The results revealed that the isolation of the RF switch without annealing was 1.4 dB at 39 GHz. As shown in Figures 6 and 7, the difference between the insertion loss and isolation for the RF switch without annealing is very small, almost the same. This represents that the RF switch without annealing has not switching effect. The edges of the membrane contact with the ground lines of CPW because of residual stress, so that the RF switch was no switching effect. However, the residual stress of the membrane was significant improvement with annealing. As shown in Figures 6 and 7, the RF switch with annealing reveals an effective switching.

Zhu *et al.* [5] proposed a RF MEMS switch with an electrothermal actuator. The switch was actuated by the electrothermal force, and it had an insertion loss of 0.5 dB at 40 GHz and an isolation of 22.5 dB at 40 GHz. A comparison to Zhu *et al.* [5], the RF switch of this work is actuated by the electrostatic force, and the response time of electrostatic switch is usually faster than that of electrothermal switch. Park *et al.* [6] presented a RF MEMS switch that was a capacitive shunt type. The switch was actuated by the comb-drive actuators, and its actuation voltage was 25 V. Comparing to Park *et al.* [6], the actuation voltage of this work is lower than that of Park *et al.* [6]. The reason is that the springs of the RF switch in this work is designed as S-shape, resulting in reducing the stiffness of springs and the actuation voltage. Kügeler *et al.* [7] developed a RF micromachined switch with piezoelectrically actuated elements. The isolation of the RF switch was 20 dB at 15 GHz, and its actuation voltage was 10 V. The piezoelectric material is not compatible with the commercial CMOS process, so it is difficult to integrate with circuitry on-a-chip. The isolation of this work exceeds that of Kügeler *et al.* [7] because the membrane of this work is increased, leading to enhance the capacitance variation of the switch during switching. Chang *et al.* [8] manufactured a micromachined switch on GaAs substrate. The actuation voltage of the switch was 26 V. The insertion loss and isolation of the switch was 0.2 dB at 10 GHz and 17 dB at 10 GHz, respectively. The micromachined switch revealed a large residual stress. In comparison to Chang *et al.* [8], the actuation voltage and isolation of this work exceed that of Chang *et al.* [8]. The reason is that the RF switch with annealing has a flat structure. The silicon substrate of this work is also lower cost than the GaAs substrate of Chang *et al.* [8].

The insertion loss and isolation are two important performances of RF switches. Several methods can enhance the performances of insertion loss and isolation, such as adopting GaAs substrate or high resistivity substrate to reduce substrate loss [9]; reducing the resistance of membrane and springs; increasing the capacitance between membrane and signal line of CPW; enhancing the inductance effect of membrane and springs [21].

## 5. Conclusions

A RF micromachined switch has been fabricated using the commercial CMOS process. The switch needed only one wet etching post-process step to release the suspended membrane and springs. The advantages of the post-process were easy execution and low cost. In addition, the post-process was compatible with the commercial CMOS process, so the RF switch has the potential for mass-production. In order to reduce the influence of residual stress for the RF switch, an annealing process was adopted. The membrane of the switch obtained an excellent flatness after the annealing at 300 °C for 30 min. The simulation results showed that the pull-in voltage of the switch was about 14 V. Experiments showed that the actuation voltage of the switch was 14 V that the value was in good agreement with the simulation value. The measurement results showed that the RF switch had an insertion loss of 0.9 dB at 35 GHz and an isolation of 21 dB at 39 GHz.
